# CEACAM1 Negatively Regulates IL-1β Production in LPS Activated Neutrophils by Recruiting SHP-1 to a SYK-TLR4-CEACAM1 Complex

**DOI:** 10.1371/journal.ppat.1002597

**Published:** 2012-04-05

**Authors:** Rongze Lu, Hao Pan, John E. Shively

**Affiliations:** 1 City of Hope Irell & Manella Graduate School of Biological Sciences, Duarte, California, United States of America; 2 Department of Immunology, Beckman Research Institute of City of Hope, Duarte, California, United States of America; Faculté de Médecine Paris Descartes, site Necker, France

## Abstract

LPS-activated neutrophils secrete IL-1β by activation of TLR-4. Based on studies in macrophages, it is likely that ROS and lysosomal destabilization regulated by Syk activation may also be involved. Since neutrophils have abundant expression of the ITIM-containing co-receptor CEACAM1 and Gram-negative bacteria such as *Neisseria* utilize CEACAM1 as a receptor that inhibits inflammation, we hypothesized that the overall production of IL-1β in LPS treated neutrophils may be negatively regulated by CEACAM1. We found that LPS treated neutrophils induced phosphorylation of Syk resulting in the formation of a complex including TLR4, p-Syk, and p-CEACAM1, which in turn, recruited the inhibitory phosphatase SHP-1. LPS treatment leads to ROS production, lysosomal damage, caspase-1 activation and IL-1β secretion in neutrophils. The absence of this regulation in Ceacam1^−/−^ neutrophils led to hyper production of IL-1β in response to LPS. The hyper production of IL-1β was abrogated by in vivo reconstitution of wild type but not ITIM-mutated CEACAM1 bone marrow stem cells. Blocking Syk activation by kinase inhibitors or RNAi reduced Syk phosphorylation, lysosomal destabilization, ROS production, and caspase-1 activation in Ceacam1^−/−^ neutrophils. We conclude that LPS treatment of neutrophils triggers formation of a complex of TLR4 with pSyk and pCEACAM1, which upon recruitment of SHP-1 to the ITIMs of pCEACAM1, inhibits IL-1β production by the inflammasome. Thus, CEACAM1 fine-tunes IL-1β production in LPS treated neutrophils, explaining why the additional utilization of CEACAM1 as a pathogen receptor would further inhibit inflammation.

## Author Summary

Pathogens often evade the immune system by directly binding to and inhibiting neutrophils, abundant white cells that accumulate at the site of infection. For example Gram-negative *Neisseria* pathogens, such as those that cause gonorrhea or meningitis, bind the neutrophil receptor CEACAM1. Gram-negative bacteria express lipopolysaccharide (LPS) that interacts with toll-like receptor-4 (TLR4) on neutrophils. Since CEACAM1 is an inhibitory receptor, we hypothesized that LPS activation of TLR4 would be inhibited. In this paper we show that this is the case and that the mechanism of LPS inhibition involves induction of a complex between the LPS receptor TLR4, CEACAM1 and an activating kinase called Syk. In the presence of CEACAM1, an inhibitory phosphatase (opposes the kinase) is recruited to the complex that prevents the activation of Syk. The net effect is the inhibition of the pathway that normally leads to the production of the pro-inflammatory cytokine IL-1β. We show that this inhibition is lost in CEACAM1 deficient neutrophils leading to hyper production of IL-1β. We think that CEACAM1 fine-tunes the normal inflammatory response at the site of infection preventing hyper-inflammation, but in the case of Gram-negative pathogens that actually bind to neutrophils, inflammation is further blunted, favoring the infectious process.

## Introduction

Neutrophils, the most abundant leukocytes, respond to and help mediate inflammation by production of chemokines and cytokines, including IL-1β. While much is know about their migration to and activation at the site of inflammation, much less is known about the regulation of their inflammatory responses once they arrive. Importantly, CEACAM1, an ITIM-containing, abundantly expressed receptor on neutrophils, is a frequently used receptor for Gram-negative pathogens that results in an inhibition of the host immune response [1], [2]. Recently, we have shown that when CEACAM1 is genetically ablated in mice, neutrophils are over-produced and over-activated during infection with the model gram positive pathogen, *Listeria monocytogenes*, resulting in accelerated mortality [3]. We hypothesized that elevated production of IL-1β (2-3-fold over wild type) in response to infection was a major cause contributing to accelerated mortality and was likely attributable directly to neutrophils lacking the inhibitory co-receptor CEACAM1. In the case of Gram-negative *Neisseria* pathogens that bind to CEACAM1, binding leads to both epithelial colonization and inhibition of the inflammatory response [4]. The high levels of CEACAM1 on neutrophils suggest that exposure to Gram-negative bacteria would lead to a more general inhibitory response, perhaps through the TLR4 pathway in response to LPS. In order to test this hypothesis in a model system, we have directly analyzed the production of IL-1β from LPS treated wild type versus Ceacam1^−/−^ neutrophils. LPS was chosen as a well-defined ligand for TLR4 signaling and for its action on neutrophils as a single agent.

IL-1β belongs to the IL-1 cytokine family and is associated with many inflammatory responses and autoinflammatory diseases [5]–[11]. Pathogen-associated molecular patterns (PAMPs) activate Toll-like-receptors (TLRs) and NF-κB signaling dependent transcription, resulting in the transcription of the IL-1β precursor, pro-IL-1β. The maturation of IL-1β is regulated by the inflammasome, a multi-molecular complex composed of pro-caspase-1, Apoptosis-associated Speck-Like Protein (ASC), and NOD like receptor (NLR) family members, including NLRP1 (NOD like receptor containing pyrin 1), NLRP3 and NLRC4 (NOD like receptor containing CARD) [7]–[8], [12]–[13]. The fully assembled inflammasome converts pro-caspase-1 into its enzymatically active form, caspase-1, which processes pro-IL-1β into IL-1β [7], [8], [13].

LPS is an endotoxin recognized by pattern recognition receptor TLR4 in myeloid cells, including macrophages and neutrophils, that induces an inflammatory response. LPS triggers the production of pro-IL-1β through the TLR-4-NF-κB pathway in macrophages and neutrophils [14]. Since LPS alone cannot activate the maturation of IL-1β in macrophages, IL-1β production in response to LPS treatment is minimal [14]. Macrophages require second signals such as ATP or crystals to activate the NLR family proteins [13]. In contrast, LPS alone can induce high levels of mature IL-1β in neutrophils [15]. Thus, the mechanism of LPS induced IL-1β production in neutrophils must differ from macrophages in some fundamental manner. Since caspase-1 deficient neutrophils fail to produce mature IL-1β upon stimulation with LPS, it is likely that the caspase-1 dependent inflammasome NLRP3 is downstream from TLR4 [15], but the detailed mechanism from TLR4 to the inflammasome is largely unknown.

In a recent study of the differential expression of NLRP3 among hematopoeitic cells, it was shown that neutrophils express similar levels of NLRP3 as resting macrophages, and the expression of NLRP3 is significantly increased in neutrophils in response to treatment with TLR ligands, including LPS [16]. The expression of ASC, another component of inflammasomes, is also upregulated in neutrophils by inflammation [17]. Since an inflammatory stimulus like LPS may activate the NLRP3 inflammasome in neutrophils, it is of interest to determine the mechanism of its regulation.

Cellular signals including ATP triggered induction of pore formation, K^+^ efflux, ROS production and lysosomal destabilization have been shown to induce NLRP3 inflammasome assembly, procaspase-1 cleavage, pro- IL-1β cleavage and IL-1β secretion in macrophages [5], [7], [9], [13], [18]–[19]. Although the molecular mechanisms of NLRP3 inflammasome activation and IL-1β secretion are still being defined, recent studies identified an upstream kinase, spleen tyrosine kinase (Syk) as a critical mediator [5], [20]–[21]. Indeed, Syk regulates both ROS production [5], [21], [22], [23] and lysosomal activity [20], [24]–[25], two major signals for NLRP3 inflammasome activation in macrophages [7], [9], [13], [19]. For example, Syk couples the NLRP3 inflammasome activation in macrophages in response to fungi and crystals [5], [20]. Syk is a non-receptor kinase usually recruited and activated by ITAM containing receptors, such as the Ig-α/β B cell receptor and FcRγ [21]. However, Syk also associates with receptors in an ITAM-independent manner [21]. In response to stimulation by TLR ligands, Syk is phosphorylated and associates with TLRs, including TLR4 and TLR9, [26]–[28]. Furthermore, Syk inhibitors attenuate TLR downstream signaling pathways [26]–[28]. In the case of human neutrophils, TLR4 directly associates with Syk and LPS stimulation increases this association [28]. Similar to neutrophils, Syk constitutively associates with TLR4 and Myd88 in human monocytes, and p-Syk associates with TLR4 after LPS treatment [29]. Although these studies place Syk in a critical downstream position associated with TLR4 signaling, they do not answer the question if activation of Syk through LPS-TLR4 leads to caspase-1 activation, ROS production, lysosomal damage and IL-1β production in neutrophils. Furthermore, since phosphorylated Syk can, in turn, be regulated by inhibitory phosphatases such as SHP1/2 [21], it is of special interest to identify co-receptors responsible for the recruitment of these inhibitory phosphatases.

In this respect, CEACAM1 (CD66a) serves as a likely candidate based on its role as a co-inhibitory molecule in the immune system [1], [30]–[36] and its abundant expression in neutrophils [3]. CEACAM1 is composed of a variable number of Ig-like extracellular domains, a trans-membrane domain and either long or short cytoplasmic domains [30]. There are 4 murine CEACAM1 isoforms, CEACAM1-2L, CEACAM1-2S, CEACAM1-4L and CEACAM1-4S based on the number of extracellular Ig-like domains and the length of the cytoplasmic tail [30]. CEACAM1 short cytoplasmic domains primarily interact with the actin-cytoskeleton [37]–[38], while the long cytoplasmic domains in addition contain two immuno-tyrosine based inhibitory motifs (ITIMs), which upon phosphorylation recruit Src homology 2 domain-containing phosphatases (SHP-1/2) [30]. Notably, recruitment of SHP-1 by the CEACAM1 long form inhibits T cell activation through dephosphorylation of Zap-70, a Syk family kinase member [33]. However, the role of CEACAM1-SHP-1 modulation of Syk activation in other immune cells and its downstream effects has been little studied.

We report here that LPS alone activates the caspase-1 inflammasome in neutrophils. LPS treated neutrophils require ROS production and lysosomal destabilization to activate the NLRP3 inflammasome, which in turn, is coupled to Syk activation. LPS triggers Syk phosphorylation and an association between p-Syk and TLR4. LPS stimulation also leads to phosphorylation of CEACAM1 on its ITIMs, followed by recruitment of SHP-1 and incorporation into the TLR4-p-Syk complex. As expected, the recruitment of SHP-1 leads to attenuated Syk activation. Ceacam1^−/−^ neutrophils exhibit elevated Syk activation, leading to augmented ROS production and lysosomal destabilization, along with caspase-1 activation and IL-1β production. Reintroduction of different CEACAM1 isoforms into Ceacam1^−/−^ neutrophils demonstrates that the inhibition provided by CEACAM1 depends on the ITIMs in CEACAM1 long form and the recruitment of SHP-1. Blocking Syk activation by the inhibitor piceatannol or reduction of Syk amounts by RNAi significantly down-regulated NLRP3 inflammasome activation in Ceacam1^−/−^ neutrophils, normalizing their IL-1β production. Therefore, our results indicate that CEACAM1 acts as a critical checkpoint in the regulation of Syk coupled NLPR3 inflammasome activation, limiting the amount of IL-1β production in neutrophils. The importance of the negative regulation of the inflammasome in neutrophils may be related to their abundant accumulation at the site of infection, where the amount of IL-1β release may need to be finely controlled. Yet, the fact that they highly express the components of the inflammasome suggests that they are capable of generating rather large amounts of IL-1β, which may contribute to runaway inflammation in the case of sepsis or diseases such as inflammatory bowel disease where neutrophils predominate and are activated by bacterial products such as LPS. Finally, the fact that potent pathogens such as *Neisseria* are able to suppress inflammation is likely due to the use of CEACAM1 as a receptor and its role in the regulation IL-1β production.

## Results

### LPS induced inflammasome activation in neutrophils depends on ROS production and lysosomal destabilization

**Figure 1:**
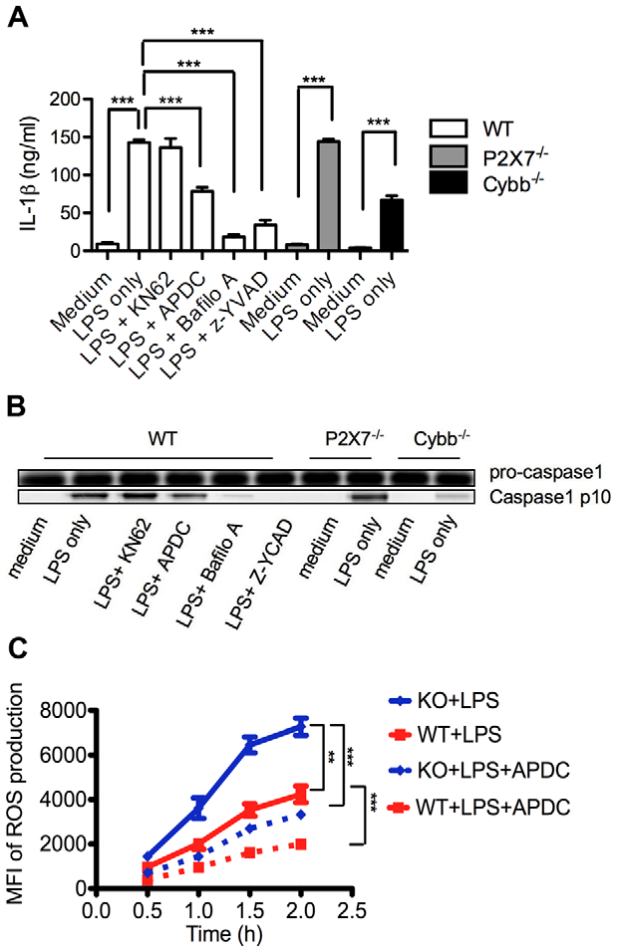
LPS induced neutrophil inflammasome activation depends on ROS production and lysosome destabilization. (**A**) IL-1β production in the supernatants of wild type (WT), P2X7^−/−^ and Cybb^−/−^ neutrophils under different treatment conditions: LPS (100 ng/ml), KN62 (2 µM, neutrophils were pre-treated for 30 minutes before LPS treatment), APDC (100 µM), bafilomycin A (125 nM), z-YVAD-fmk (1 mM). (**B**) Immunoblot analysis showing caspase-1 activation of WT, P2X7^−/−^ and Cybb^−/−^ neutrophils under different treatment conditions. (**C**) ROS production by LPS treated WT and CEACAM1^−/−^ neutrophils with or without APDC (100 µM) as measured by MFI of fluorescent probe H2DCFDA using FACS. Data are representative of 3 different experiments (**B**) and p values (**A** and **C**) were calculated by a 2-tailed T-test, **0.001<p≤0.01 ,***p≤0.001.

Since previous studies show LPS alone is sufficient for neutrophil IL-1β production, we were interested in determining whether the caspase-1 inflammasome is activated and the mechanisms for its activation. Consistent with previous studies, LPS alone activated IL-1β production in neutrophils (**Figure 1A–B**), but not in macrophages that required the additional signals ATP or alum crystals (**Figure S1A**). Since ATP and potassium efflux have been implicated in the secretion of IL-1β in macrophages [18], we treated LPS primed neutrophils with KN62, an antagonist of the P2X7 receptor, the receptor largely responsible for mediating these ATP effects [39]. Although we did observe ATP release from LPS treated neutrophils (**Figure S1B**), KN62 failed to block caspase-1 activation or IL-1β production (**Figure 1A–B**). Furthermore, LPS fully stimulated IL-1β production in P2X7 deficient neutrophils (**Figure 1A–B**), confirming the dispensability of exogenous ATP for LPS induced IL-1β production. We also examined the role of K^+^ efflux by blocking potassium channels with glibenclamide [5], a treatment that showed no measureable reduction of IL-1β production (data not shown), excluding the involvement of K^+^ efflux in neutrophil LPS induced inflammasome activation. To test whether ROS production was necessary for inflammasome activation in neutrophils, we treated WT neutrophils with (2R, 4R)-4-aminopyrrolidine-2, 4-dicarboxylate (APDC), an inhibitor of NADPH-oxidase-dependent ROS production [5]. Indeed, APDC inhibited LPS stimulated ROS production in neutrophils by 70% (**Figure 1C**), and IL-1β production by 45% (**Figure 1A–B**). To further test the role of ROS production on the inflammasome, neutrophils from gp91phox-deficient (Cybb^−/−^) mice [19] were treated with LPS. In these neutrophils less caspase-1 activation and IL-1β production were observed (**Figure 1A–B**), indicating that ROS production significantly mediates neutrophil inflammasome activation. Lysosomal destabilization commonly refers to both the release of cathepsin B and the inability of the lysosomal compartment to retain a low pH necessary for its lytic activities. When neutrophil lysosomal acidification was inhibited with bafilomycin A [5], [19], caspase-1 activation and IL-1β secretion were significantly reduced (**Figure 1A–B**), demonstrating the critical role of lysosomal destabilization. Besides showing that caspase-1 was activated in LPS treated neutrophils, we also blocked caspase-1 activation using the caspase-1 inhibitor z-YVAD-fmk [5], [19], resulting in reduced caspase-1 activation and IL-1β production (**Figure 1A–B**), and suggesting that the caspase-1 containing inflammasome is mainly responsible for neutrophil IL-1β production. We conclude that LPS induced ROS production and lysosomal destabilization, which in turn, activated the inflammasome in neutrophils.

### LPS induced inflammasome activation is coupled to Syk activation in neutrophils

**Figure 2:**
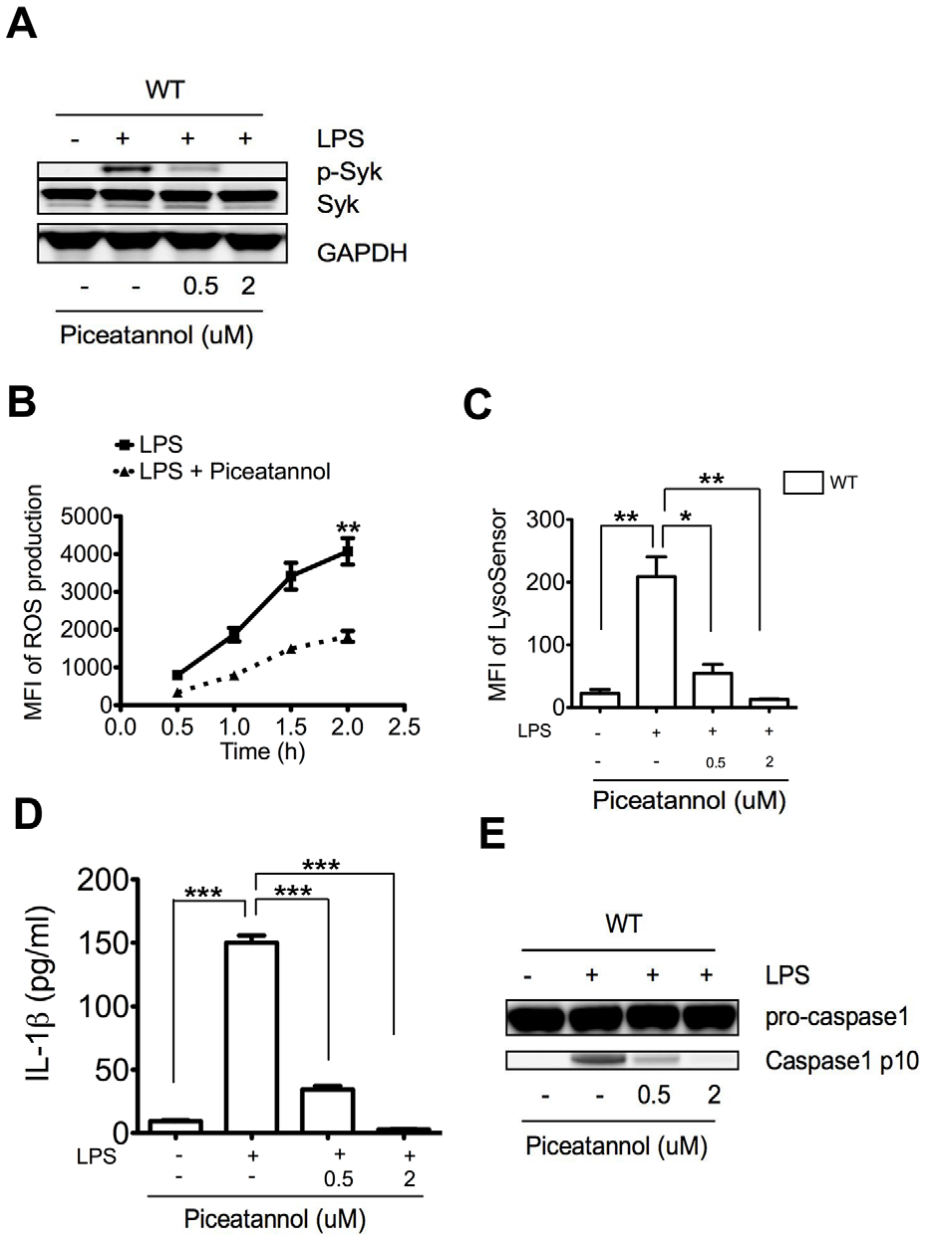
Syk activation is coupled to LPS induced neutrophil inflammasome activation. (**A**) Immunoblot analysis of p-Syk (Y525/526), Syk and GAPDH of neutrophils before and after LPS treatment (100 ng/ml) in the presence of the Syk inhibitor piceatannol. (**B**) ROS production by LPS treated neutrophils with or without piceatannol treatment (0.5 µM) as measured by MFI of fluorescent probe H2DCFDA using FACS. (**C**) Quantification of lysosome destabilization of neutrophils with or without piceatannol treatment (0.5 µM) as measured by MFI of LysoSensor Green using FACS. (**D**) IL-1β production in the supernatants of LPS treated WT neutrophils with or without piceatannol treatment. (**E**) Immunoblot analysis showing caspase-1 activation of LPS treated neutrophils with or without piceatannol treatment. Data are representative of 3 different experiments (**A** and **E**) and p values (**D** and **E**) were calculated by a 2-tailed T-test *0.01<p≤0.05,**0.001<p≤0.01,***p≤0.001.

Since Syk regulates macrophage inflammasome activation by modulation of ROS production and lysosomal destabilization [5], [20]–[21], we asked if Syk is similarly coupled to LPS triggered neutrophil inflammasome activation. LPS induced Syk phosphorylation (p-Syk 525/526) in neutrophils was down regulated in a dose dependent manner by piceatannol (**Figure 2A**), a specific Syk inhibitor [20], [26]. Piceatannol also decreased LPS triggered neutrophil ROS production (**Figure 2B**) as shown by reduction of mean fluorescence intensity (MFI) of the ROS sensitive fluorescent probe 2′7′-dichlorofluorescin diacetate (H2DCFDA) [5] and lysosomal destabilization as shown by reduction of MFI of LysoSensor Green (**Figure 2C**), a pH-sensitive dye which fluoresces only after encountering acidic environments [19]. Correspondingly, piceatannol treatment also down-regulated caspase-1 activation and IL-1β production (**Figure 2D–E**). Unlike crystal induced lysosomal destabilization in macrophages, which requires phagocytosis [9], [19]–[20], LPS triggered lysosomal destabilization in neutrophils was phagocytosis independent, because treatment of neutrophils with the phagocytosis inhibitor cytochalasin D, did not affect LPS triggered lysosomal destabilization or capase-1 activation (data not shown). Thus, neutrophils, the most abundant subset of granulocytes, require Syk, ROS, and lysosomal destabilization as a general inflammasome activation mechanism in response to LPS.

### Genetic ablation of CEACAM1 leads to elevated p-Syk and inflammasome activation in neutrophils

**Figure 3:**
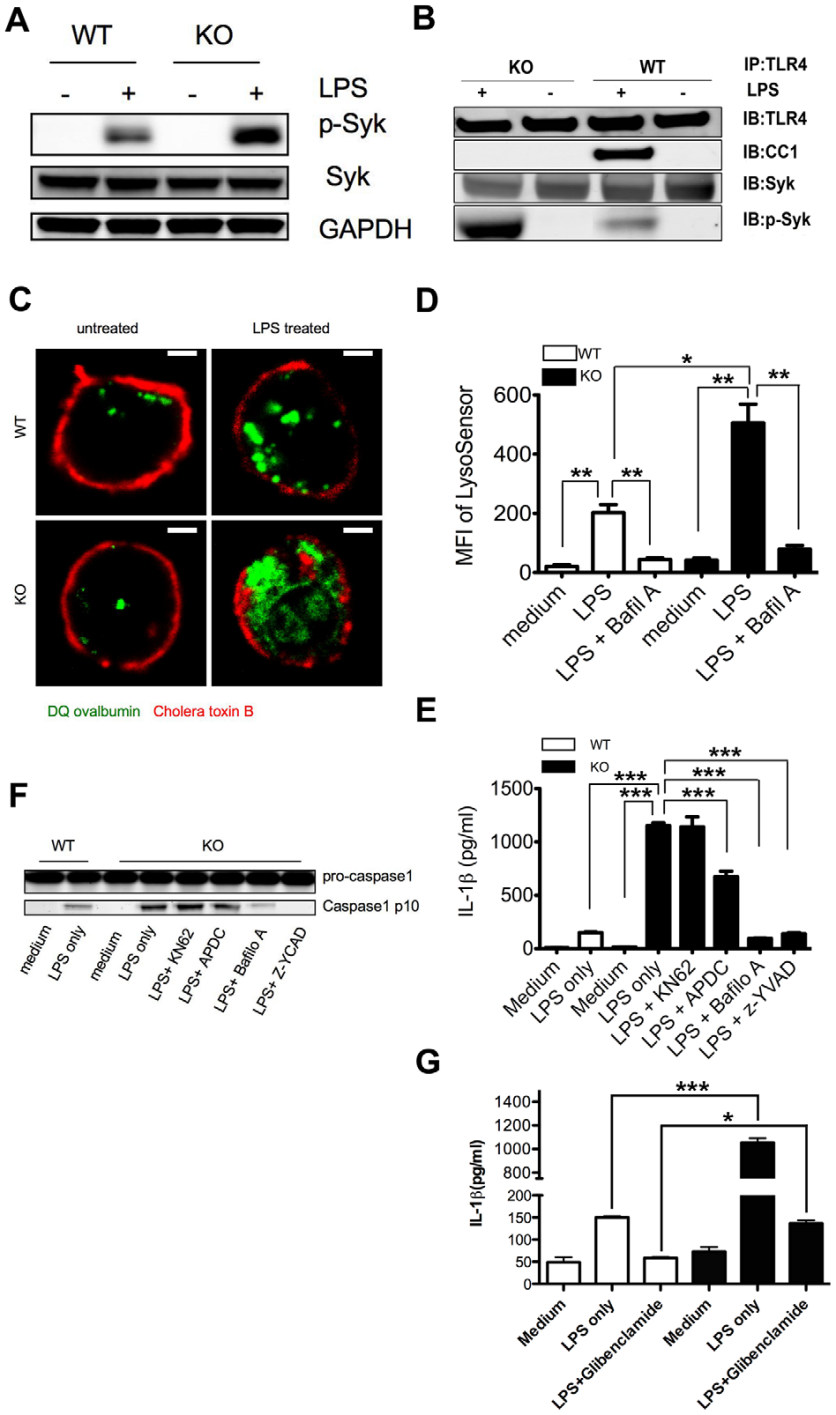
Loss of CEACAM1 leads to elevated Syk activation and enhanced inflammasome activation. (**A**) Immunoblot analysis of WT and Ceacam1^−/−^ (KO) neutrophils with p-Syk (Y525/526), Syk and GAPDH antibodies. (**B**) Immunoblot analysis of TLR4, Syk, p-Syk and CEACAM1 in WT and Ceacam1^−/−^ (KO) neutrophils with or without LPS treatment (100 ng/ml) after immunoprecipitation with TLR4 antibody. (**C**) Confocal microscopy showing LPS induced lysosomal destabilization in WT and Ceacam1^−/−^ neutrophils (DQ-Ovalbumin, 10 mg/ml; green) with or without LPS (100 ng/ml), cell membranes were stained with fluorescent cholera toxin B-subunit (red). (**D**) Quantification of lysosomal destabilization of WT and Ceacam1^−/−^ neutrophils as measured by MFI of LysoSensor Green using FACS. (**E**) IL-1β production in the supernatants of LPS treated WT and Ceacam1^−/−^ neutrophils under different treatment conditions. (**F**) Immunoblot analysis showing caspase-1 activation of LPS treated WT and Ceacam1^−/−^ neutrophils under different treatment conditions. (**G**) IL-1β production in the supernatants of LPS treated WT neutrophils with or without glibenclamide treatment (250 µM). Data are representative of 3 different experiments and p values (**C**) were calculated by a 2-tailed T-test *0.01<p≤0.05,**0.001<p≤0.01,***p≤0.001.

Since Syk positively regulates LPS induced inflammasome activation in neutrophils, it is of interest to study the regulatory mechanism of Syk activation. CEACAM1 is a likely candidate due to its high expression in neutrophils and since CEACAM1 was shown to negatively regulate the related kinase Zap-70 in activated T cells [33]. We hypothesized that neutrophils lacking CEACAM1 would have unregulated Syk mediated inflammasome activation leading to high IL-1β production. Indeed, genetic ablation of CEACAM1 led to dramatically higher levels of p-Syk in Ceacam1^−/−^ neutrophils compared with WT neutrophils (**Figure 3A**). We also compared global tyrosine phosphorylation for WT versus Ceacam1^−/−^ neutrophils after stimulation with LPS and found no obvious differences (data not shown), demonstrating that it is important to look at kinases using phosphotyrosine specific antibodies. To study the mechanism of the activation of Syk, we performed immunoprecipitation (IP) studies with neutrophils from Ceacam1^−/−^ and WT mice. While Syk was co-IPed with TLR4 before and after treatment with LPS, pSyk co-IPed with Syk and TLR4 only in response to LPS treatment and this association increased in Ceacam1^−/−^ compared to wild type neutrophils (**Figure 3B**). We also IPed TLR4 and tested its association with Dectin, a lectin associated with an ITAM response to bacteria with engagement of Syk [23], [40]. We found that LPS stimulation did not trigger the association of Dectin to the TLR4 complex (data not shown). Thus, LPS is a specific trigger of Syk activation, and the activated form remains associated with TLR4. Furthermore, when TLR4 was IPed after LPS treatment, the inhibitory co-receptor CEACAM1 was found associated with TLR4 (**Figure 3B**). Thus, both CEACAM1 and pSyk only associate with TLR4 when activated by LPS. The mechanism by which CEACAM1 negatively regulates p-Syk in this complex will be explored later (see below). In addition, a previous study has shown that CD16/32 is not involved with LPS-stimulated TLR4 signals in neutrophils [41]. As a negative control, we IPed CD16/32 followed by immunoblotting with 4G10 on WT vs Ceacam1^−/−^ neutrophils after LPS stimulation and found a small amount of activation of this ITAM containing FcR, but to the same degree in WT and Ceacam1^−/−^ neutrophils (data not shown).

Elevated Syk activation was associated with enhanced ROS production and was greatest for LPS treated Ceacam1^−/−^ neutrophils (**Figure 1C**). In addition, elevated p-Syk correlated with augmented lysosomal destabilization in Ceacam1^−/−^ neutrophils. To demonstrate this on isolated cells, WT and Ceacam1^−/−^ neutrophils were incubated with DQ ovalbumin, a lysosomal uptake marker, [19] with or without LPS. As expected, DQ ovalbumin was restricted to lysosomes in resting stage WT and Ceacam1^−/−^ neutrophils (**Figure 3C**), but spread into the cytosol upon lysosomal rupture in LPS treated neutrophils (**Figure 3C**). Moreover, this effect was greatest in Ceacam1^−/−^ neutrophils due to enhanced inflammasome activation. To quantify lysosomal destabilization, neutrophils were stained with acridine orange, a fluorescent dye that is highly fluorescent in environmentally acidic lysosomes but is shifted to lower fluorescence in disrupted lysosomes [19]. As expected, we observed a higher percentage of a lower fluorescent peak in Ceacam1^−/−^ neutrophils versus WT neutrophils after LPS treatment (**Figure S2**). Consistently, the increased MFI of the LysoSensor Green dye in Ceacam1^−/−^ neutrophils confirmed augmented lysosomal destabilization in these cells compared to WT cells (**Figure 3D**). Due to augmented ROS production and lysosomal destabilization, Ceacam1^−/−^ neutrophils exhibited significantly more IL-1β production (7 fold, **Figure 3E**) and caspase-1 activation (**Figure 3F**) upon LPS treatment compared to WT neutrophils. Gibenlamide, also known as glyburide, an inhibitor of the NLRP3 inflammasome, blocked IL-1ß production in both WT and CEACAM1 deficient neutrophils. (**Figure 3G**). These data demonstrate that the production of IL-1ß in response to LPS is through the NLRP3 inflammasome in neutrophils. Since genetic or inhibitor studies may affect the apoptotic status of neutrophils, we also stained the neutrophils with annexin V. The results of annexin V staining indicate that apoptosis was minimal (<7%) in both WT and Ceacam1^−/−^ neutrophils before and after LPS treatment (data not shown).

Although the increased IL-1β production in Ceacam1^−/−^ neutrophils could be due to increased transcription of IL-1β, there was no difference of IL-1β mRNA amounts in WT and Ceacam1^−/−^ neutrophils as measured by quantitative real-time PCR (**Figure S3A**), indicating a similar degree of TLR-NF-κB signaling in WT and Ceacam1^−/−^ neutrophils. This result also suggests that the elevated amounts of p-Syk were not caused by enhanced TLR-NF-κB signaling. In addition, we tested whether various IL-1β inhibitors affect the production of TNF-α, another TLR-NF-κB dependent cytokine. We found no significant difference in the levels of TNF-α among treatment with the various inhibitors in both WT and Ceacam1^−/−^ neutrophils. (**Figure S3B**) Similar to WT neutrophils, Ceacam1^−/−^ neutrophils also depended on ROS and lysosomal destabilization for inflammasome activation since they responded similarly to WT neutrophils to KN62, APDC and bafilomycin A as manifested by caspase-1 activation and IL-1β production (**Figure 3E–F**). Unlike murine neutrophils, murine macrophages only express moderate amounts of CEACAM1 [3], and WT and Ceacam1^−/−^ bone marrow derived macrophages (BMDMs) secreted similar amounts of IL-1β upon stimulation by LPS with ATP or Alum crystals (**Figure S1A**), suggesting that the genetic ablation of CEACAM1 does not affect macrophage IL-1β production.

### LPS triggered IL-1β production is partially dependent on cathepsin B in WT and Ceacam1^−/−^ neutrophils

**Figure 4:**
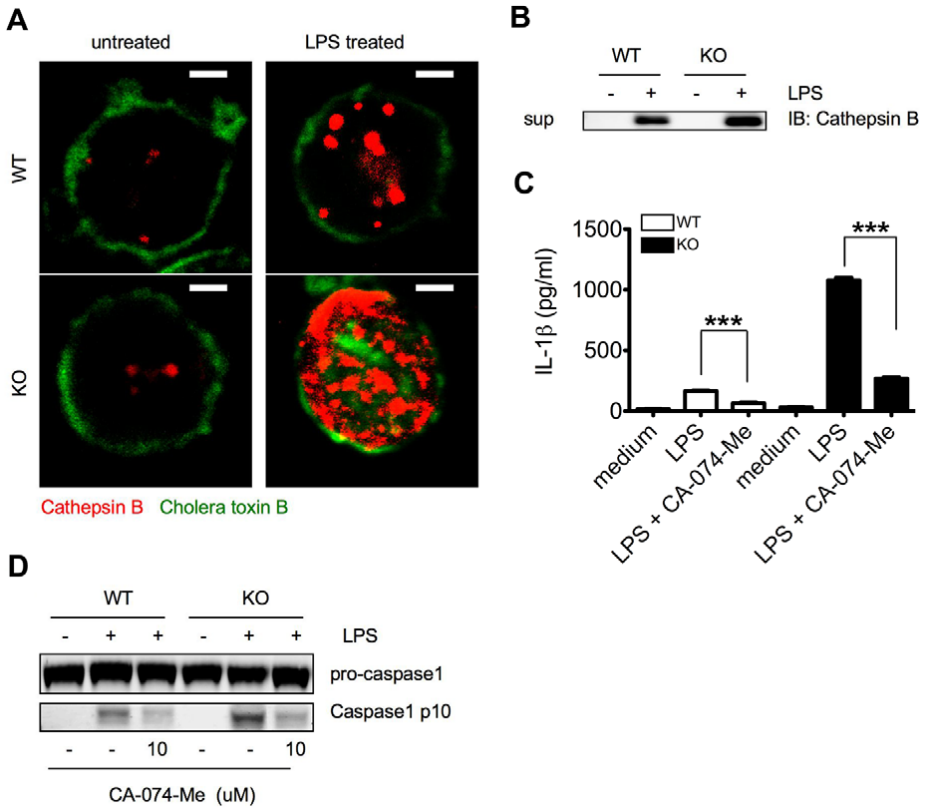
LPS triggered IL-1β production in WT and Ceacam1^−/−^ neutrophils is partially dependent on cathepsin B. (**A**) Confocal microscopy showing cathepsin B activity in LPS treated WT and Ceacam1^−/−^ (KO) neutrophils. (**B**) Immunoblot analysis showing cathepsin B amounts in the supernatants of LPS treated WT and Ceacam1^−/−^ neutrophils. (**C**) IL-1β production in the supernatants of LPS treated WT and Ceacam1^−/−^ neutrophils after treatment with CA-074-Me. (**D**) Immunoblot analysis showing caspase-1 activation of LPS treated WT and Ceacam1^−/−^ neutrophils after CA-074-Me treatment. Data are representative of 3 different experiments (**A**, **B** and **D**) and p values (**C**) were calculated by a 2-tailed T-test ***p≤0.001.

Previous studies on murine macrophages stimulated by crystals or hemozoin identified the lysosomal protease cathepsin B as an activator of the NLRP3 inflammasome, since cathepsin B inhibitors partially blocked activation [3], [6], [9], [19]–[20]. To determine if a similar mechanism may operate in LPS treated neutrophils, we examined cathepsin B activity in WT and Ceacam1^−/−^ neutrophils. LPS treated Ceacam1^−/−^ neutrophils exhibited significantly elevated cathepsin B activity compared to WT neutrophils (**Figure 4A**), with higher amounts of cathepsin B in the supernatant of Ceacam1^−/−^ neutrophils (**Figure 4B**). Moreover, the cathepsin B inhibitor CA-074-Me partially blocked WT and Ceacam1^−/−^ neutrophil caspase-1 activation and IL-1β production (**Figure 4C–D**). Although the mechanism of cathepsin B mediated NLRP3 inflammasome activation is unclear, we may conclude that LPS triggered cathepsin B release is at least partially responsible for NLRP3 activation in neutrophils.

### CEACAM1 down-regulates Syk activation through ITIM phosphorylation and recruitment of SHP-1

**Figure 5:**
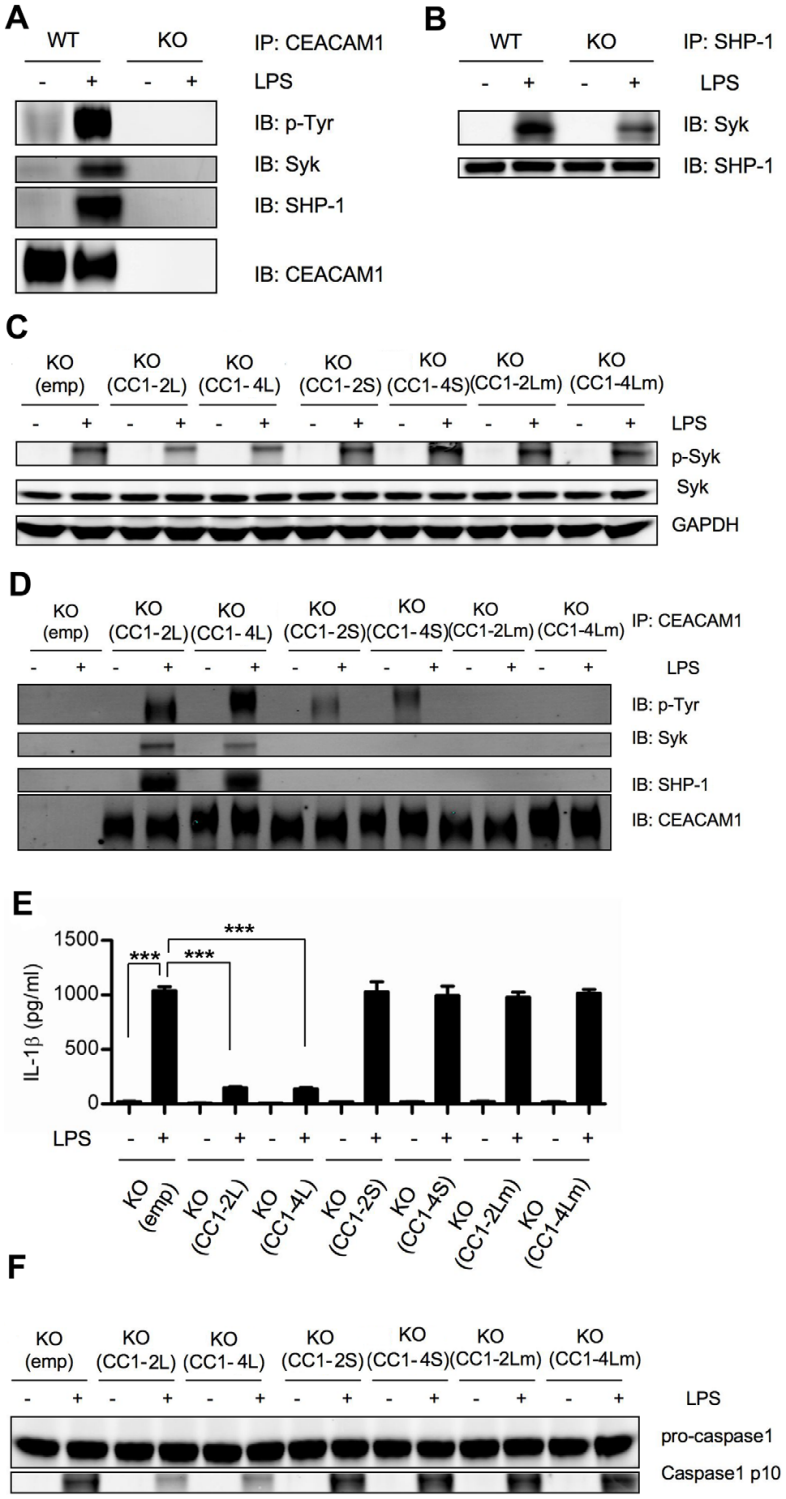
CEACAM1 down-regulates Syk activation through ITIM recruitment of SHP-1. (**A**) Immunoblot analysis of p-Tyr, Syk, SHP-1 and CEACAM1 in WT and Ceacam1^−/−^ (KO) neutrophils with or without LPS treatment (100 ng/ml) after IP with anti-CEACAM1 antibody. (**B**) Immunoblot analysis of Syk, SHP-1 in WT and Ceacam1^−/−^ neutrophils with or without LPS after IP with anti-SHP-1 antibody. (**C**) Immunoblot analysis of p-Syk, Syk and GAPDH in neutrophils from Ceacam1^−/−^ mice reintroduced with empty vector (KO/(emp)), CEACAM1-2L (KO/(CC1-2L)), CEACAM1-4L (KO/(CC1-4L)), CEACAM1-2S (KO/(CC1-2S)), CEACAM1-4S (KO/(CC1-4S)), ITIMs mutated CEACAM1-2L (KO/(CC1-2 Lm)) and ITIMs mutated CEACAM1-4L (KO/(CC1-4 Lm)) with or without LPS treatment. (**D**) Immunoblot analysis of the p-Tyr, Syk, SHP-1 and CEACAM1 in neutrophils from KO/(emp), KO/(CC1-2L), KO/(CC1-4L), KO/(CC1-2S), KO/(CC1-4S), KO/(CC1-2 Lm) and KO/(CC1-4 Lm) chimeras after IP with CEACAM1 antibody. (**E**) IL-1β production in the supernatants of LPS treated neutrophils from KO/(emp), KO/(CC1-2L), KO/(CC1-4L), KO/(CC1-2S), KO/(CC1-4S), KO/(CC1-2 Lm) and KO/(CC1-4 Lm) chimeras. (**F**) Immunoblot analysis showing caspase-1 activation of LPS treated neutrophils KO/(emp), KO/(CC1-2L), KO/(CC1-4L), KO/(CC1-2S), KO/(CC1-4S), KO/(CC1-2 Lm) and KO/(CC1-4 Lm) chimeras. Data are representative of 3 different experiments and p values (**E**) were calculated by a 2-tailed T-test ***p≤0.001.

CEACAM1 functions as a co-inhibitory receptor through phosphorylation of its ITIMs and the recruitment of SHP-1 [1], [3], [30]–[35]. Above we showed that p-Syk is elevated in Ceacam1^−/−^ neutrophils upon LPS stimulation and that TLR4 is associated with both pSyk and CEACAM1 (**Figure 3B**), suggesting that CEACAM1 is also a negative regulator of Syk activation. Since the likely mechanism of inhibition involves phosphorylation of CEACAM1's ITIMs followed by recruitment of SHP-1, we IPed CEACAM1 and performed immunoblot analysis with anti-phosphotyrosine, anti-Syk and anti-SHP-1. Significant tyrosine phosphorylation of CEACAM1 (**Figure 5A**) and significant amounts of Syk were co-IPed in LPS treated WT neutrophils (**Figure 5A**), thus indicating that CEACAM1 was phosphorylated on its ITIMs and that Syk was physically associated with CEACAM1 after LPS treatment. As predicted, SHP-1 was recruited to phosphorylated CEACAM1 in an LPS dependent manner (**Figure 5A**). Furthermore, when SHP-1 was IPed, it was recruited more strongly to LPS treated WT rather than Ceacam1^−/−^ neutrophils (**Figure 5B**), in agreement with the expectation that association of SHP-1 with Syk is enhanced in WT neutrophils when CEACAM1 is present.

To prove that the inhibition provided by CEACAM1 in vivo relies on phosphorylation of its ITIMs and SHP-1 recruitment, we reintroduced CEACAM1-2L, CEACAM1-4L, CEACAM1-2S, CEACAM1-4S, ITIM mutated CEACAM1-2L (CEACAM1-2Lm) and ITIM mutated CEACAM1-4L (CEACAM1-4Lm) into Ceacam1^−/−^ bone marrow stem cells using GFP containing retroviral vectors, and then injected GFP positive cells back into lethally irradiated Ceacam1^−/−^ recipients. Two months later, we isolated neutrophils and examined their phenotypes (**scheme shown in Figure S4**). We found that compared to neutrophils recovered from mice reconstituted with empty vectors transduced Ceacam1^−/−^ bone marrow stem cells, p-Syk amounts were reduced in neutrophils receiving CEACAM1 long isoforms, but not in those receiving the short isoforms (**Figure 5C**). Furthermore, this down-regulation was abrogated by mutation of the ITIMs in CEACAM1 long isoforms (**Figure 5C**). Consistently, only CEACAM1 long isoforms containing intact ITIMs were able to associate with Syk as shown by IP analysis with CEACAM1 antibody (**Figure 5D**). As expected, the down-regulation of Syk activation by restoration of CEACAM1 long isoforms in Ceacam1^−/−^ neutrophils attenuated ROS production and lysosomal destabilization (data not shown), leading to reduced IL-1β production and caspase-1 activation (**Figure 5E–F**).

These results are similar to previous studies on T-cells in which activation of T-cells led to induction of CEACAM1 [34] followed by phosphorylation of CEACAM1 and recruitment of SHP-1, which in turn, led to down activation of the Syk-related kinase ZAP-70 [33]. The main difference is that CEACAM1 is constitutively expressed in neutrophils, thus maintaining a relatively low level of Syk mediated inflammasome activation prior to and after LPS activation.

### Reduction of SHP-1 amounts by RNAi leads to elevated Syk activation and augmented inflammasome activation

**Figure 6:**
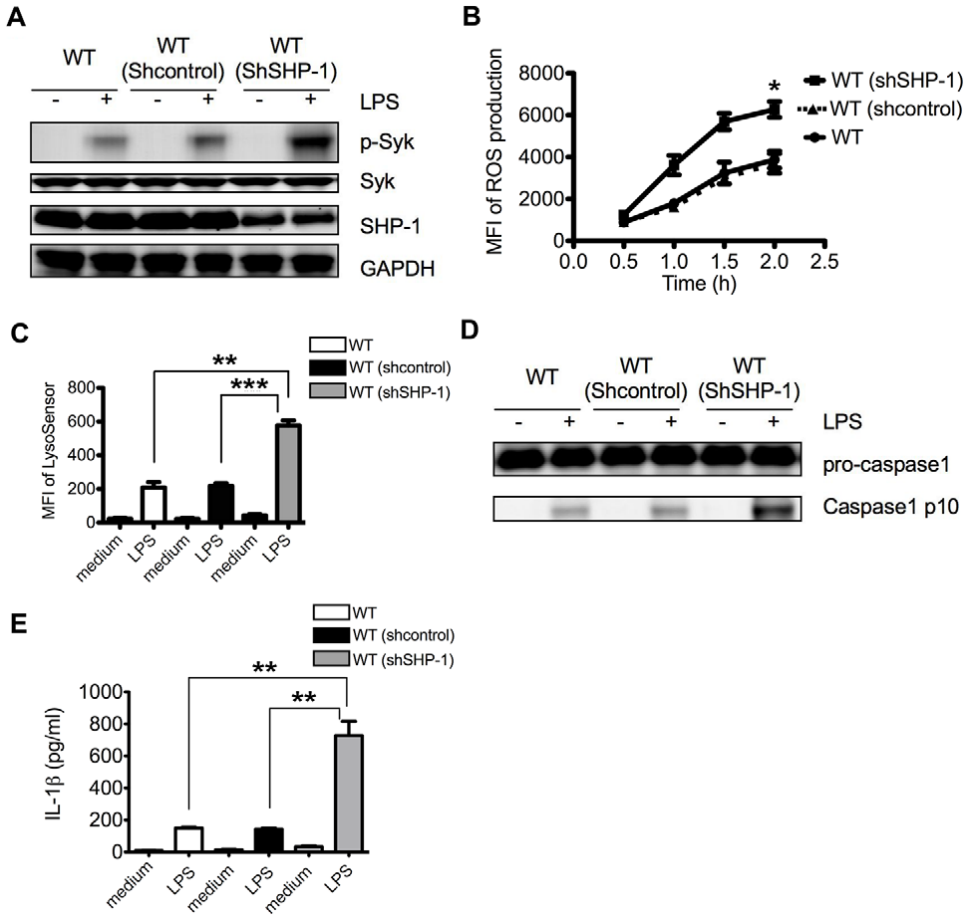
RNAi reduction of SHP-1 leads to elevated Syk activation and augmented inflammasome activation. (**A**) Immunoblot analysis of p-Syk, Syk, SHP-1 and GAPDH of LPS treated neutrophils from WT mice reconstituted with shRNA control retroviral vector transduced WT BM cells (WT/(shcontrol)), WT mice reconstituted with shSHP-1 retroviral vector transduced WT BM cells (WT/(shSHP-1)) compared with WT mice. (**B**) ROS production by LPS treated neutrophils from WT mice, WT/(shcontrol) and WT/(shSHP-1) chimera measured by MFI of fluorescent probe H2DCFDA using FACS. (**C**) Quantification of lysosome destabilization of LPS treated neutrophils from WT mice, WT/(shcontrol) and WT/(shSHP-1) chimera measured by MFI of LysoSensor Green using FACS. (**D**) Immunoblot analysis showing capase-1 activation of LPS treated neutrophils from WT mice, WT/(shcontrol) and WT/(shSHP-1). (**E**) IL-1β production of LPS treated neutrophils from WT mice, WT/(shcontrol) and WT/(shSHP-1) chimera. Data are representative of 3 different experiments (**A** and **D**) and p values (**B, C** and **E**) were calculated by a 2-tailed T-test *0.01<p≤0.05,**0.001<p≤0.01,***p≤0.001.

To further confirm that CEACAM1 regulation of Syk mediated inflammasome activation depends on SHP-1 recruitment, we reduced the amount of endogenous SHP-1 with retroviral vectors containing GFP and shRNA against SHP-1. We transduced WT bone marrow stem cells with shRNA containing retroviral vectors and injected GFP selected cells into lethally irradiated WT recipients. We then recovered neutrophils after 2 months of bone marrow reconstitution. Immunoblot analysis confirmed an efficient reduction of SHP-1 mRNA amounts (**Figure 6A**), and showed elevated Syk phosphorylation compared with neutrophils isolated from WT mice reconstituted with control shRNA retroviral vector transduced WT bone marrow stem cells (**Figure 6A**). The elevated Syk activation correlated with augmented neutrophil ROS production (**Figure 6B**) and lysosomal destabilization (**Figure 6C**). Correspondingly, we observed elevated caspase-1 activation and IL-1β production after RNAi mediated reduction of SHP-1 amounts (**Figure 6D–E**). These data demonstrate the indispensable role of SHP-1 in the down-regulation of Syk coupled NLRP3 inflammasome activation in neutrophils.

### Src family kinase Lyn acts upstream of Syk and CEACAM1

Syk is usually activated by phosphorylation with a Src kinase [21]. For example, Src family kinase Lyn phosphorylates Syk in macrophages [20]. To determine if Lyn is associated with Syk in neutrophils, we IPed Lyn and found that Syk was specifically pulled down in LPS treated WT neutrophils (**Figure S5A**). Importantly, CEACAM1 was also pulled down by anti-Lyn antibody (**Figure S5A**), suggesting that Lyn may phosphorylate both Syk and CEACAM1 in LPS treated neutrophils. This result is consistent with previous studies on human neutrophils in which Lyn was shown to phosphorylate CEACAM1 [42]. In order to further confirm that a Src kinase regulates Syk activation in neutrophils, we treated LPS primed neutrophils with the Src kinase inhibitor PP2, a treatment that blocked Syk phosphorylation in a dose dependent manner (**Figure S5B**). Correspondingly, PP2 also significantly blocked LPS induced ROS production (data not shown) and lysosomal destabilization (data not shown), as well as the downstream caspase-1 activation and IL-1β production (**Figure S5C–D**).

### Blocking Syk activation or reduction of Syk amounts leads to attenuated inflammasome activation and IL-1β production in Ceacam1^−/−^ neutrophils

**Figure 7:**
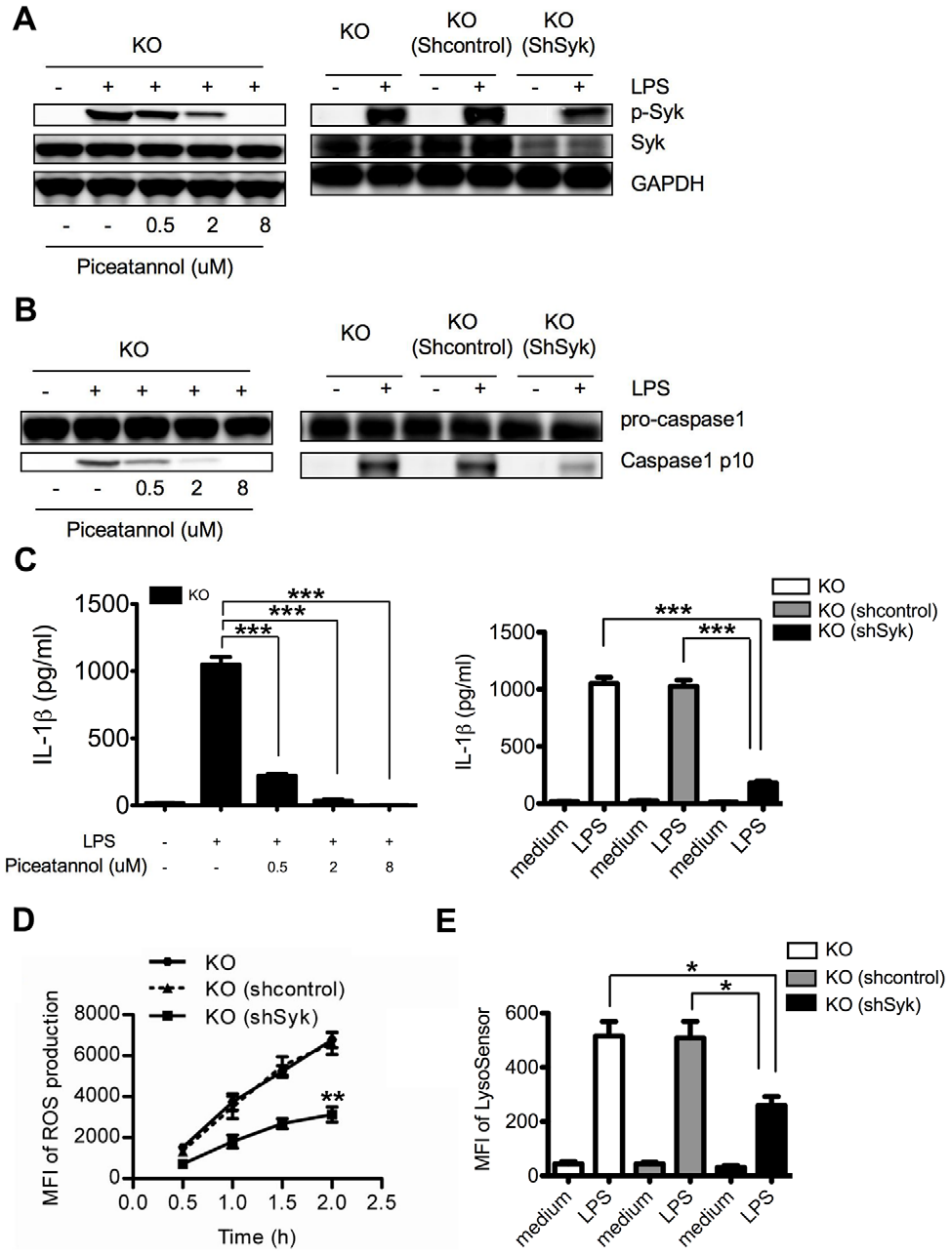
Blocking Syk activation or reduction of Syk amounts significantly inhibits inflammasome activation in Ceacam1^−/−^ neutrophils. (**A**) Left, immunoblot analysis of p-Syk, Syk, GAPDH of LPS treated Ceacam1^−/−^ (KO) neutrophils with or without piceatannol; Right, immunoblot analysis of p-Syk, Syk, GAPDH of LPS treated neutrophils from Ceacam1^−/−^ mice reconstituted with shRNA control retroviral vector transduced Ceacam1^−/−^ BM cells (KO/(shcontrol)), CEACAM1^−/−^ mice reconstituted with shSyk retroviral vector transduced Ceacam1^−/−^ BM cells (KO/(shSyk)) compared with KO mice. (**B**) Left, immunoblot analysis of caspase-1 activation of LPS treated CEACAM1^−/−^ neutrophils with or without piceatannol; right, immunoblot analysis of caspase-1 activation of LPS treated neutrophils from Ceacam1^−/−^ mice, KO/(shcontrol) and KO/(shSyk) chimera. (**C**) Left, IL-1β production of LPS treated neutrophils from Ceacam1^−/−^ mice with or without piceatannol; right, IL-1β production of LPS treated neutrophils from KO mice, KO/(shcontrol) and KO/(shSyk) chimera. (**D**) ROS production by LPS treated neutrophils from Ceacam1^−/−^ mice, KO/(shcontrol) and KO/(shSyk) chimera measured by MFI of fluorescent probe H2DCFDA using FACS. (**E**) Quantification of lysosome destabilization of LPS treated neutrophils from Ceacam1^−/−^ mice, KO/(shcontrol) and KO/(shSyk) chimera measured by MFI of LysoSensor Green using FACS. Data are representative of 3 different experiments (**A** and **B**) and p values (**C, D** and **E**) were calculated by a 2-tailed T-test *0.01<p≤0.05,**0.001<p≤0.01,***p≤0.001.

To further test if elevated Syk activation is indeed responsible for augmented inflammasome activation in Ceacam1^−/−^ neutrophils, we inactivated Syk with a kinase inhibitor and reduced endogenous Syk amounts by RNAi treatment with the expectation of reducing inflammasome activation and IL-1β production comparable to WT levels. Indeed, piceatannol treatment of LPS stimulated Ceacam1^−/−^ neutrophils blocked Syk phosphorylation in a dose dependent manner (**Figure 7A**), and also dramatically inhibited LPS induced ROS production (data not shown), lysosomal damage (data not shown), caspase-1 activation (**Figure 7B**) and IL-1β production (**Figure 7C**). Moreover, reduction of Syk with RNAi normalized the amounts of p-Syk in LPS stimulated Ceacam1^−/−^ neutrophils to amounts comparable to WT neutrophils (**Figure 7A**). As expected, this treatment significantly attenuated the amount of ROS production (**Figure 7D**), lysosomal damage (**Figure 7E**), caspase-1 activation (**Figure 7B**) and IL-1β production (**Figure 7C**) in LPS stimulated Ceacam1^−/−^ neutrophils. We conclude that elevated Syk activation was mechanistically responsible for increased inflammasome activation in Ceacam1^−/−^ neutrophils because either inhibition of Syk activation or reduction of endogenous Syk, normalized caspase-1 activation and IL-1β production.

## Discussion

CEACAM1 (CD66a) has long been used as a marker for neutrophils due to its abundant expression and further up-regulation during activation [43]. Since it is an ITIM-containing type 1 transmembrane protein, its main functional role in lymphocytes generates inhibitory signals [1], [30]. The fact that many pathogens, including *Neisseria*, utilize CEACAM1 as a receptor on neutrophils results in a potent inhibition of the inflammatory response [1], [2], [44]. Although the mechanism of the inhibition has been studied at the level of CEACAM1's ITIM phosphorylation, the downstream effects on key components of the inflammatory response such as the inflammasome have not been studied. In fact, recent attention has turned to the role of CEACAM3, an ITAM-containing member of the CEA gene family, as the chief regulator of a positive inflammatory response in *Neisseria* infected neutrophils [4]. Yet, the predominantly high levels of CEACAM1 on neutrophils suggest that inhibition would be the main outcome of LPS mediated gram-negative infections. Thus, we speculated that the inhibition was mediated by the LPS signaling pathway and not solely due to the direct binding of the bacteria to CEACAM1 or CEACAM3. Notably, the CEACAM3 response has been directly linked to Syk activation, which according to our findings would be inhibited by CECAM1 inhibition of the LPS-TLR4 pathway.

While inflammasome activation has been intensively studied in macrophages [7]–[8], [12]–[13], [18]–[19], the possibility that similar mechanisms apply to the more abundant neutrophils has not been comprehensively explored. In contrast to macrophages which need two signals to activate inflammasomes, in neutrophils, we found that LPS alone not only induced the production of pro-IL-1β, but also induced its processing to mature pro-IL-1β. We found that LPS stimulated neutrophils utilized ROS production and lysosomal destabilization as signals to activate the inflammasome. LPS induced TLR4 and the Src kinase Lyn association with Syk, a kinase previously found coupled to ROS production and lysosomal damage in PAMP stimulated macrophages [9], [19]–[20]. The inhibitory co-receptor CEACAM1 was recruited to TLR4 after LPS treatment of neutrophils and recruited SHP-1, that in turn, inhibited Syk phosphorylation. As a result, Ceacam1^−/−^ neutrophils exhibited enhanced Syk phosphorylation and elevated ROS production and lysosomal damage in response to LPS. Correspondingly, LPS treated neutrophils from Ceacam1^−/−^ mice showed elevated caspase-1 activation and IL-1β production, demonstrating that CEACAM1 negatively regulates NLRP3 inflammasome activation.

The role of ROS in inflammasome activation has been recently investigated [45], [46]. Since both macrophages and neutrophils utilize ROS to kill bacteria via activation of NADPH oxidase, it was originally postulated that the source of ROS in macrophages was NADPH oxidase [47]. However, subsequent studies showed that gp91phox (a component of the NADPH oxidase complex) deficient mice produced normal amounts of IL-1β in response to various inflammasome activators [48]. When gp91phox deficient (Cybb^−/−^) neutrophils were treated with LPS in our study, IL-1β production was reduced by 50% suggesting that, at least in neutrophils, NADPH oxidase was an important, but not the sole source of ROS. Further comparison between neutrophils and macrophages are necessary to resolve these apparent differences.

Syk mediates inflammasome activation in macrophages by fungi and crystals [5], [20]–[21]. We found that activation of the inflammasome by LPS in neutrophils provides a direct link between activation of TLR4 and Syk. We also found that Src kinase associated with Syk, and that blocking Src kinase activity abrogated Syk activation, indicating that a Src kinase (Lyn) is responsible for phosporylation of Syk. Although ITAM containing receptors are responsible for the classical activation of Syk, Syk can also be activated through ITAM-independent signals [21]. For example, LPS and other TLR ligands activate Syk [26]–[28]. TLR4 interacts with Syk constitutively in neutrophils and peripheral blood mononuclear cells, and upon LPS treatment, TLR4 associates with p-Syk [28]–[29]. The TLR4 cytoplasmic domain has a putative motif, which upon phosphorylation, can be recognized by the SH2 domain of Syk [28], [49]. Our data in murine neutrophils agree with previous studies in human neutrophils that show that TLR4 associates with p-Syk upon LPS treatment [28].

Although elevated amounts of p-Syk induced more ROS production and lysosomal damage in Ceacam1^−/−^ neutrophils, they did not affect pro-IL-1β or TNF-α transcription which is a distinct pathway dependent on TLR4-NFκB signaling. This result is consistent with previous studies that show Syk activation is not directly involved in TLR4-NF-κB signaling [50] since normal amounts of TNF-α and IL-6 are induced by LPS treatment in Syk^−/−^ models [51]. However, Syk activation is critical for production of TNF-α in response to C-type lectin mediated fungal recognition in macrophages [5]. Thus, there may be a receptor mediated pathway or cell-type difference in the immune response to different PAMPs in respect to the role of Syk.

Similar to previous studies in T cells where CEACAM1 associates with Zap-70 after T cell activation [33], we also found that CEACAM1 associated with the related kinase Syk in LPS treated neutrophils. Since CEACAM1 functions as an ITIM-SHP-1 dependent co-inhibitory receptor in various types of immune cells [1], [31]–[36], we tested whether CEACAM1 down-regulates neutrophil inflammasome activation in an ITIM-SHP-1 dependent manner in vivo by reintroducing different CEACAM1 isoforms into Ceacam1^−/−^ mice. Consistent with this hypothesis, we found that only CEACAM1 long isoforms restored normal Syk activation, inflammasome activation and IL-1β production, while mutating the ITIMs of CEACAM1 long isoforms abrogated this restoration. Also IP analysis using SHP-1 antibody pulled down Syk in LPS treated WT and Ceacam1^−/−^ neutrophils, indicating that SHP-1 can directly dephosphorylate Syk. Notably, there was still an association between SHP-1 and Syk in Ceacam1^−/−^ neutrophils although this association was significantly reduced in the absence of CEACAM1, indicating that other ITIM containing receptors may also recruit SHP-1 and dephosphorylate Syk. The dependency on SHP-1 was also demonstrated by RNAi mediated reduction of endogenous SHP-1 in WT mice, a treatment that led to enhanced p-Syk amounts, caspase-1 activation and IL-1β in LPS treated neutrophils. These results are consistent with the previous observation that SHP-1 deficient (moth-eaten) mice produced more IL-1β in response to PAMPs compared to wild type mice [52]–[53]. Our study is the first to identify CEACAM1 as the key co-receptor responsible for recruitment of SHP-1 to Syk followed by inhibition of the inflammasome.

Due to the higher numbers of neutrophils compared to monocytes and macrophages in the bone marrow, there is a greater requirement for negative regulation of the inflammasome in response to inflammatory signals such as LPS. In agreement with this idea, we found a ten-fold higher level of expression of CEACAM1 in murine BM neutrophils compared to monocytes and macrophages [3]. Thus, the levels of CEACAM1, as well as its activation and recruitment to TLR4, may be major regulators of the inflammasome in both neutrophils and monocytes.

**Figure 8:**
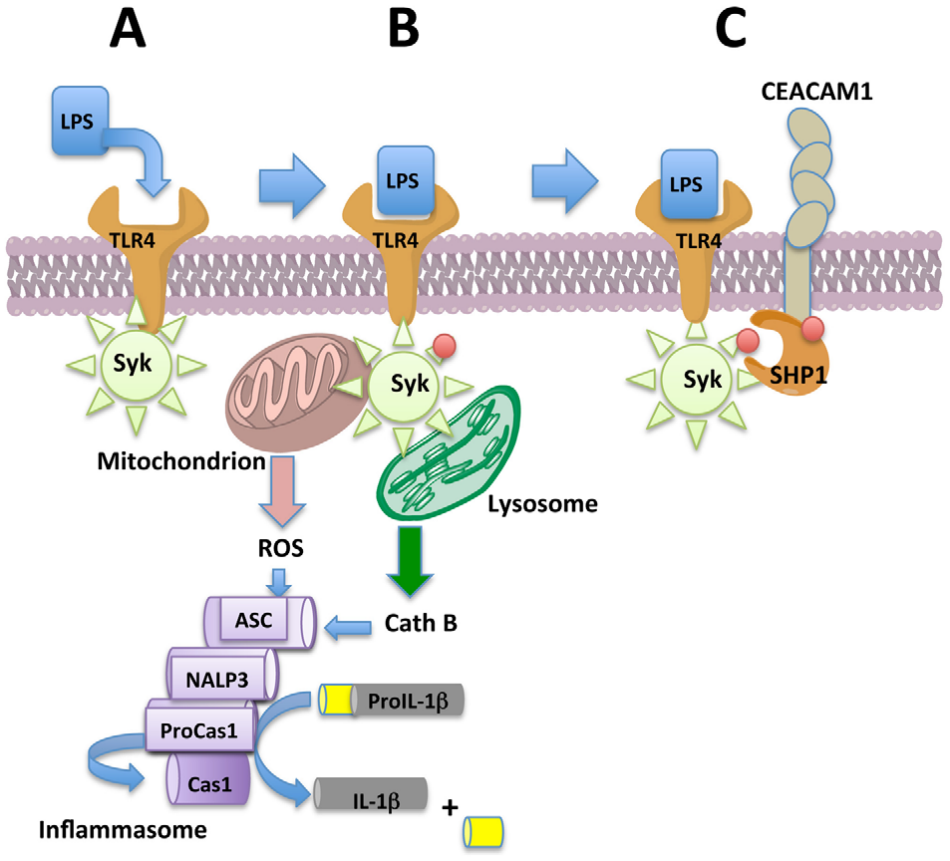
Model for the inhibition of the inflammasome in neutrophils by CEACAM1. (**A**) LPS binds to TLR (the usual downstream effects such as activation of NFκB are not shown for clarity). (**B**) The complex recruits and activates Syk (pSyk) which in the absence of CEACAM1 fully activates the inflammasome that includes ROS production from the mitochondrion and cathepsin B from the lysosome. The activated inflammasome converts pro-caspase-1 to active caspase-1, which in turn converts pro-IL-1β to active IL-1β. (**C**) In the presence of CEACAM1, both Syk and CEACAM1 are phosphorylated when LPS binds to TLR4. CEACAM1 recruits SHP1 via its phosphorylated ITIM. SHP1 dephosphorylates pSyk, reducing the production of ROS and lysosome disruption, which in turn, reduces the activity of the inflammasome.

In summary, our results demonstrate that LPS triggers TLR4-Syk mediated inflammasome activation in neutrophils. In addition, CEACAM1, an ITIM containing co-receptor, fine-tunes the activation of the pro-inflammatory kinase Syk and its attendant immune response in neutrophils (**Figure 8**). We have identified a critical feedback loop provided by CEACAM1 that down-regulates the inflammasome activation and IL-1β production in neutrophils. This study suggests that manipulation of CEACAM1 inhibition may provide clinical applications for treatment of dangerous IL-1β dependent inflammatory responses and auto-inflammatory diseases. This study also reveals a mechanism by which pathogens that utilize CEACAM1 as a receptor can inhibit inflammation.

## Materials and Methods

### Mice

No human research was performed in this study. This study was carried out in strict accordance with the recommendations of the Guide for the Care and use of Laboratory Animals of the National Institutes of Health. The protocol (number 08017) was approved by the Institutional Animal Care and Use Committee (IACUC) of the City of Hope, an AAALAC approved facility (assurance number A3001-01). Ceacam1^−/−^ mice were generously provided by Nicole Beauchemin (McGill University, Montreal, Canada). WT C57/B6 mice were purchased from National Cancer Institute. P2X7^−/−^ C57/B6 and Cybb^−/−^ C57/B6 were purchased from Jackson Laboratory. Mice 7–12 weeks old were used for all the experiments.

### Cell sorting and FACS analysis

Neutrophils were purified using antibody labeling and magnetic beads depletion as described below. Briefly, total bone marrow cells were flushed out using PBS plus 2% FBS. Red blood cells were lysed using red blood cell lysis buffer, total cells labeled with biotin conjugated anti-Ly-6C, anti-B220, anti-CD3, anti-Ter119, anti-F4/80, anti-CD11c, anti-ICAM, anti-NK1.1 and anti-CD19, washed and incubated with anti-biotin microbeads followed by auto magnetic-activated cell sorting (AutoMACS). FACS analysis for measuring ROS production and lysosome destabilization was performed as previously described [5], [19]. For measuring ROS production, purified neutrophils were treated with LPS (Sigma, catalogue No. L2880-10MG, impurities<3%) for indicated times in the presence of fluorescent probe 2′7′-dichlorofluorescin diacetate (H2DCFDA), cells were permeabilized (BD Cytofix/Cytoperm) and MFI of FITC was measured as indicator of ROS production. For measuring neutrophil lysosomal destabilization, neutrophils were treated with LPS for 2 hours and then LysoSensor was added immediately before FACS analysis. Neutrophils were also incubated with acridine orange together with LPS for 2 hours, and cells with loss of lysosomal function were measured as loss of acridine orange staining (PE-Cy5 channel). The FACS were performed on FACS Canto II (BD Biosciences)

### Quantitative Real-time PCR

RNA extractions from neutrophils and BMPCs were performed using Trizol (Invitrogen). Reverse transcription was performed (QIAGEN) followed by real-time on Bio-Rad IQ5 Real-time Detection system. IL-1β mRNA was detected using the following primers: 5′-CAGGCAGGCAGTATCACTCA-3′ and 5′-AGCTCATATGGGTCCGACAG-3′. The IL-1β mRNA level was normalized to HPRT expression using the following primers 5′-GCTGGTGAAAAGGACCTCT-3′ and 5′-CACAGGACTAGAACACCTGC-3′. The amplification parameters were initiated at 95°C for 5 min, then 95°C for 30 s, 57°C for 30 s, and 72°C for 30 s for 40 cycles, followed by 7 min at 72°C for the final extension.

### Confocal microscopy

To observe neutrophil lysosome destabilization, neutrophils were incubated for 2 hours with DQ ovalbumin (10 mg/ml) together with LPS (100 ng/ml), then cells were permeabilized and stained with Alexa fluor 647 cholera toxin B-subunit for 30 minutes. Cathepsin B activity was measured using Cathepsin B detection kit according to instructions (Enzo lifesciences). Confocal microscopy was performed on LSM 510 Meta Inverted 2 Photon.

### Construction and generation of retroviral vectors

XhoI and EcoRI restriction sites were generated at the 5′ and 3′ ends of CEACAM1-2L, 4L, 2S or 4S cDNAs using the following primers: 5′-GAGTCTCGAGATGGAGCTGGCCTCAGCACATC-3′ and 5′-GAGTGAATTCTCACTTCTTTTTTACTTCTGAATAAAC-3′, and subsequently cloned into retroviral vector (MSCV-EGFP) which was kindly provided by Zuoming Sun (Department of Immunology, Beckman Research Institute of City of Hope). The ITIM mutations on CEACAM1-2L and 4L were generated using QuikChange XL Site-Directed Mutagenesis Kit (Stratagene) with the following primers: 5′-CAAGGTGGATGACGTCGCAGCCACTGTCCTGAACTTCAAT-3′ and 5′-ATTGAAGTTCAGGACAGTGGCTGCGACGTCATCCACCTTG-3′ (for the first Tyr), and 5′-CCTTCTTCTCCAAGAGCCACAGAAACAGTTGCTTCAGAAGTAAAAAAG-3′ and 5′-CTTTTTTACTTCTGAAGCAACTGTTTCTGTGGCTCTTGGAGAAGAAGG-3′ (for the second Tyr). The shSyk control vector, shSyk vector (pGFP-V-RS) and ShSHP-1 vector (pGFP-V-RS) were purchased from Origene, and the target sequence of shSyk vector is 5′-CTTTGTCGGTGGCTCACAACAGGAAGGCA-3′, and the target sequence of shSHP-1 vector is 5′-TTGTGCGTGAGAGTCTCAGCCAACCTGGT-3′. All retroviral vectors were generated in Phoenix-Eco packaging cell line. For MSCV-EGFP based vectors, viral titers of the supernatants from the stable virus-producing cells were determined in NIH 3T3 cells by FACS. Both Phoenix and NIH3T3 cell lines were kindly provided by Dr. Zuoming Sun (city of Hope).

### Retroviral transduction of bone marrow stems cells

5-FU (5 mg/mouse) was injected i.p. into Ceacam1^−/−^ mice and bone marrow stem cells were collected 5 days after injection. Stem cells were expanded in 10% FBS DMEM medium plus 20 ng/ml IL-3, 50 ng/ml IL-6 and SCF for 24 hours, followed by retroviral transductions. For selecting MLCV based empty vector, CEACAM1-2L, 4L, 2S, 4S, 2 Lm and 4 Lm transduced cells, GFP positive cells were sorted 48 to 72 hours after transduction. For selecting MSCV based shRNA control vector, shSyk vector and shSHP-1 vector, puromycin (1 ug/ml) resistant cells were collected 7 days later after transduction. Collected cells were then injected i.v. into lethally irradiated Ceacam1^−/−^ or WT recipients (1–5×10^5^ cells/mouse). Two months later, phenotypes of neutrophils from reconstituted recipients were characterized.

### Immunoblot and immunoprecipitation

For immunoblots, 5×10^6^ neutrophils with/without LPS treatment (100 ng/ml) for 30 minutes to measure Syk phosphorylation, and 2 hours for measuring caspase-1 activation. Cells were lysed using RIPA buffer (Sigma) supplemented with 4 mM Na_3_VO_4_, 50 mM NaF and 1 mM PMSF. Total cell extracts were examined by immunoblot using corresponding antibodies for specific proteins as previously described [34]. Immuno-precipitation was performed as previously described [34]. Briefly, 50×10^6^ neutrophils with/without LPS treatment (100 ng/ml) were lysed using 1% NP40 supplemented with 4 mM Na_3_VO_4_, 50 mM NaF and 1 mM PMSF, the total cell lysate was pre-cleared with isotype control murine or rabbit IgG, and then anti-CEACAM1 or SHP-1 antibody was added and rotated at 4°C overnight. The next day, protein G agarose was added and rotated at 4°C for 4 hours, washed, and run on SDS gels for immunoblot analysis.

### ELISA

Neutrophils were treated with or without LPS for 4 hours and supernatants collected. BMDMs were treated with LPS for 4 hours then with ATP (5 mM) or Alum (200 ug/ml) for 1 hour and supernatants collected. TNF-α, IL-1β levels were measured with ELISA kits according to manufacturers' instructions (BioLegend).

## Supporting Information

Figure S1 **IL-1β from bone marrow derived macrophages (BMDM) and ATP from neutrophils of WT and Ceacam1^−/−^ mice.** (**A**) BMDM were treated as shown. (**B**) ATP release from neutrophils over time.
(TIF)

Figure S2 **Flow analysis of LPS treated WT and Ceacam1^−/−^ neutrophils with acridine orange.** Loss of lysosomal staining with acridine orange (excitation 488 nm, emission 650–690 nm) shows increase of the lower fluorescent peak after LPS treatment.
(TIF)

Figure S3 **IL-1β mRNA levels and TNFα protein levels in WT and Ceacam1^−/−^ neutrophils.** (**A**) IL-1β mRNA levels in LPS treated neutrophils over time. (**B**) Protein levels of TNFα from neutrophils treated as shown.
(TIF)

Figure S4 **Experimental design for retroviral transduction and bone marrow reconstitution.**
(TIF)

Figure S5 **Src family kinase Lyn acts upstream of Syk and CEACAM1.** (**A**) Immunoblot analysis showing Syk, CEACAM1 and Lyn in WT and KO neutrophils with or without LPS treatment (100 ng/ml) after immunoprecipitation with anti-Lyn antibody. (**B**) Immunoblot analysis showing p-Syk and Syk in WT and KO neutrophils with LPS treatment. (**C**) ELISA analysis showing IL-1 β production by LPS treated WT and KO neutrophils with or without PP2 treatment. (**D**) Immunoblot analysis showing caspase-1 activation of LPS treated WT and KO neutrophils with or without PP2 treatment.
(TIF)
