# Supplementary figures and images for: CEACAM1 Negatively Regulates IL-1β Production in LPS Activated Neutrophils by Recruiting SHP-1 to a SYK-TLR4-CEACAM1 Complex
Source: PLoS Pathog. 2012 Apr 5;8(4):e1002597. doi: 10.1371/journal.ppat.1002597 (PMC3320586; doi:10.1371/journal.ppat.1002597)

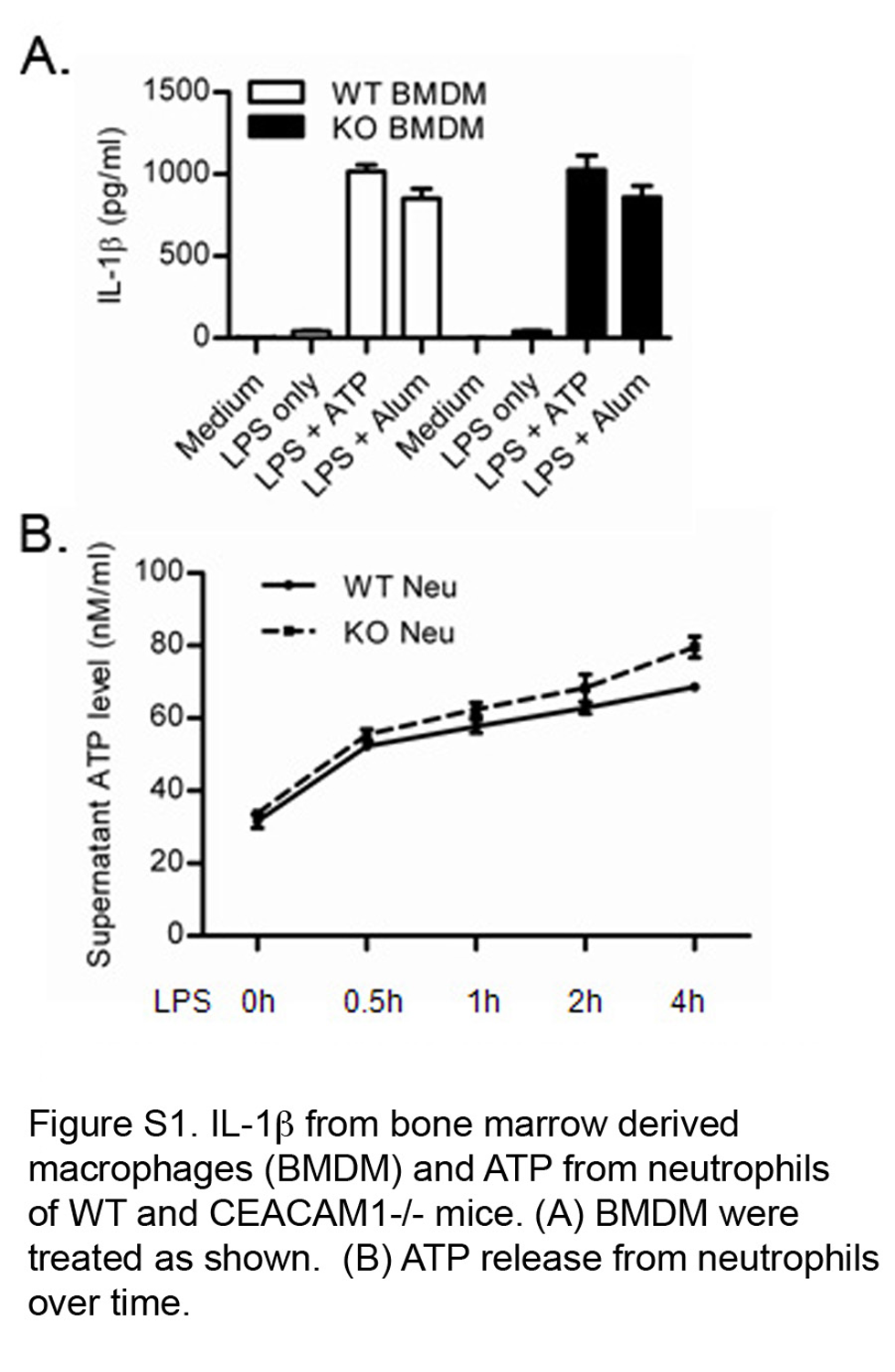

Supplement: Figure S1 — IL-1β from bone marrow derived macrophages (BMDM) and ATP from neutrophils of WT and Ceacam1−/− mice. (A) BMDM were treated as shown. (B) ATP release from neutrophils over time. (TIF) [file ppat.1002597.s001.tif]

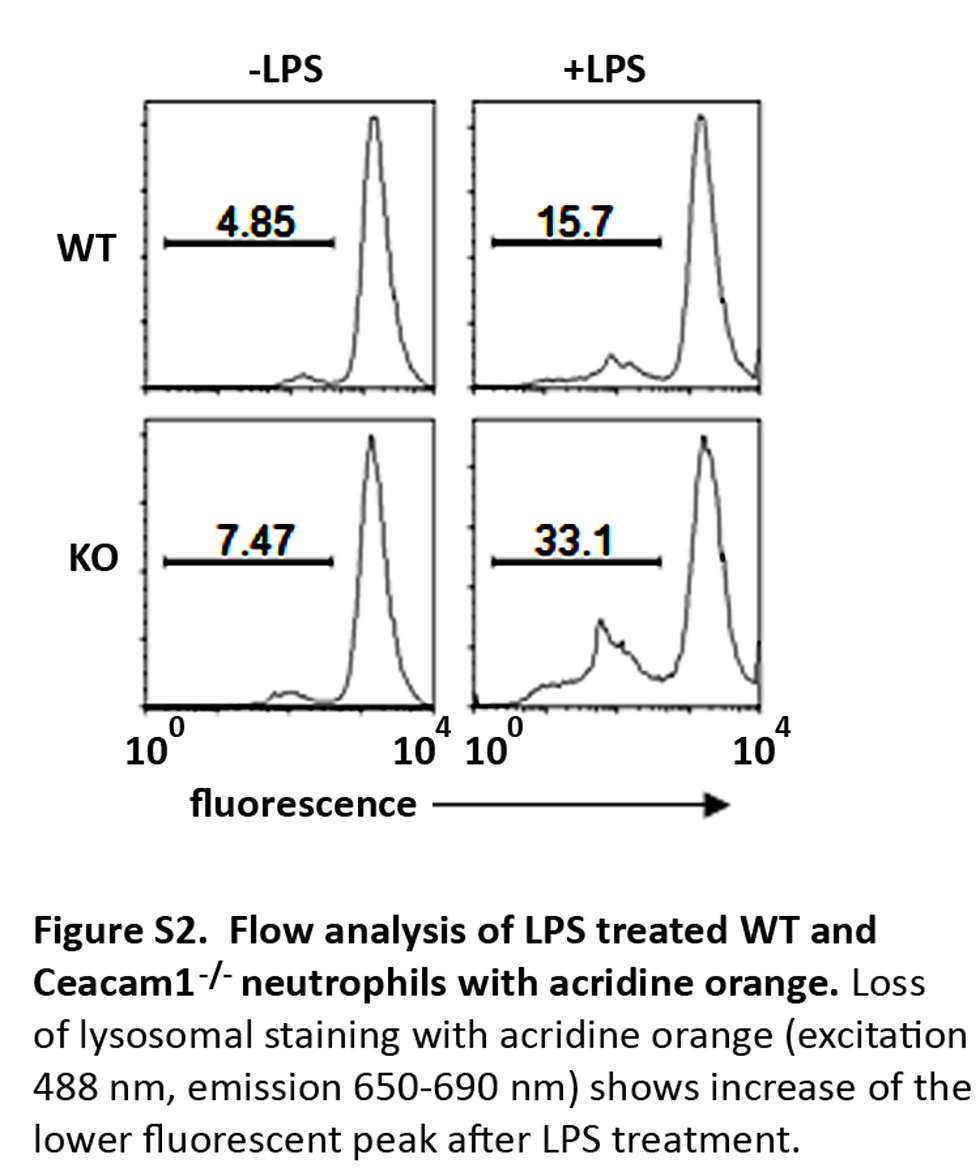

Supplement: Figure S2 — Flow analysis of LPS treated WT and Ceacam1−/− neutrophils with acridine orange. Loss of lysosomal staining with acridine orange (excitation 488 nm, emission 650–690 nm) shows increase of the lower fluorescent peak after LPS treatment. (TIF) [file ppat.1002597.s002.tif]

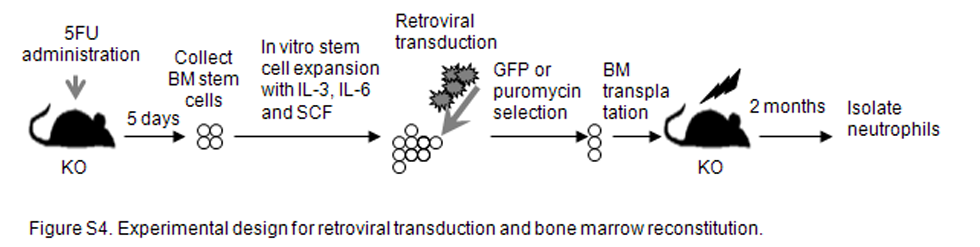

Supplement: Figure S4 — Experimental design for retroviral transduction and bone marrow reconstitution. (TIF) [file ppat.1002597.s004.tif]

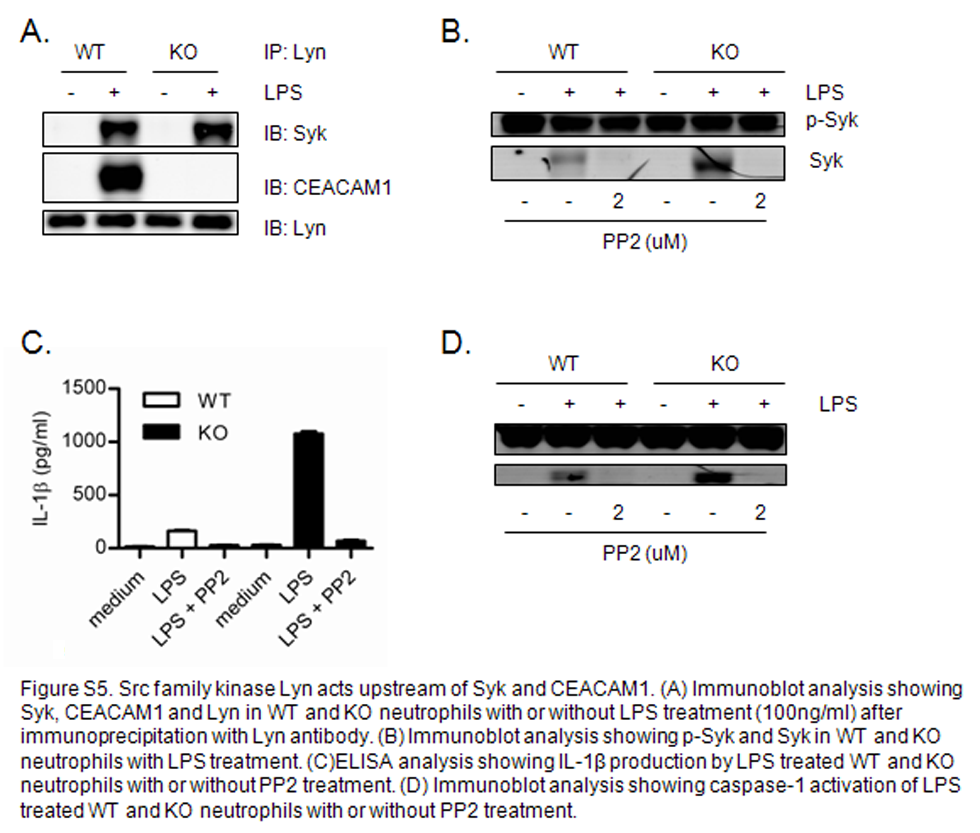

Supplement: Figure S5 — Src family kinase Lyn acts upstream of Syk and CEACAM1. (A) Immunoblot analysis showing Syk, CEACAM1 and Lyn in WT and KO neutrophils with or without LPS treatment (100 ng/ml) after immunoprecipitation with anti-Lyn antibody. (B) Immunoblot analysis showing p-Syk and Syk in WT and KO neutrophils with LPS treatment. (C) ELISA analysis showing IL-1 β production by LPS treated WT and KO neutrophils with or without PP2 treatment. (D) Immunoblot analysis showing caspase-1 activation of LPS treated WT and KO neutrophils with or without PP2 treatment. (TIF) [file ppat.1002597.s005.tif]
